# Supplementary material for: Development of R7BP inhibitors through cross-linking coupled mass spectrometry and integrated modeling
Source: Commun Biol. 2019 Sep 13;2:338. doi: 10.1038/s42003-019-0585-1 (PMC6744478; doi:10.1038/s42003-019-0585-1)
Supplement: Supplementary file 1 — Supplementary Information [file 42003_2019_585_MOESM1_ESM.pdf]

Supplementary Figures

```
RGS6   MAQGSGD-QRAVGVADPEESSPNMIVYCKIEDIITKMQDDKTGGVPIRTV
RGS7   MAQGNNYGQTSNGVAD---ESPNMLVYRKMEDVIARMQDEKN-GIPIRTV
RGS9   MTIRHQG-Q-------Q--YRPRMAFLQKIEALVKDMQNPET-GVRMQNQ
RGS11 MAAGPAP-P-------PGRPRAQMPHLRKMERVVVSMQDPDQ-GVKMRSQ
      *:                    ..*    *:* ::  **: .  *: ::.

RGS6   KSFLSKIPSVVTGTDIVQWLMKNLSIEDPVEAIHLGSLIAAQGYIFPISD
RGS7   KSFLSKIPSVFSGSDIVQWLIKNLTIEDPVEALHLGTLMAAHGYFFPISD
RGS9   RVLVTSVPHAMTGSDVLQWIVQRLWISS-LEAQNLGNFIVRYGYIYPLQD
RGS11 RLLVTVIPHAVTGSDVVQWLAQKFCVSE-EEALHLGAVLVQHGYIYPLRD
      : ::: :* ..:*:*::**: :.: :..   ** :** .:.  **::*: *

RGS6  H-VLTMKDDGTFYRFQAPYFWPSNCWEPENTDYAIYLCKRTMQNKARLEL
RGS7  H-VLTLKDDGTFYRFQTPYFWPSNCWEPENTDYAVYLCKRTMQNKARLEL
RGS9  PKNLILKPDGSLYRFQTPYFWPTQQWPAEDTDYAIYLAKRNIKKKGI—L
RGS11 PRSLMLRPDETPYRFQTPYFWTSTLRPAAELDYAIYLAKKNIRKRGT--L
         * :: * : ****:****.:     . : ***:**.*:.::::.   *

RGS6   ADYEAENLARLQRAFARKWEFIFMQAEAQVKIDRKKDKTERKILDSQERA
RGS7   ADYEAESLARLQRAFARKWEFIFMQAEAQAKVDKKRDKIERKILDSQERA
RGS9   EEYEKENYNFLNQKMNYKWDFVIMQAKEQYRAGKERNKADRYALDCQEKA
RGS11 VDYEKDCYDRLHKKINHAWDLVLMQAREQLRAAKQRSKGDRLVIACQEQT
       :** :    *:: :   *::::***. * :  :::.* :*  : .**::

RGS6   FWDVHRPVPGCVNTTEMDIRKCRRLKNPQKVKKSVYGVTEESQAQSPVHV
RGS7   FWDVHRPVPGCVNTTEVDIKKSSRMRNPHKTRKSVYGLQNDIRSHSPTHT
RGS9  YWLVHRCPPGMDNVLDYGLD---RVTNPNEVKVN----------------
RGS11 YWLVNRPPPGAPDVLEQGPG---RGSCA-ASRVL----------------
      :* *:*  **  :. : .     *   .   :

RGS6   LSQPIRKTTKEDIRKQITFLNAQIDRHCLKMSKVAESLIAYTEQYVEYDP
RGS7   PTPETKPPTEDELQQQIKYWQIQLDRHRLKMSKVADSLLSYTEQYLEYDP
RGS9   -----QKQTVVAVKKEIMYYQQALMRSTVKSSVSLGGIVKYSEQFSSNDA
RGS11 -----MTKSADFHKREIEYFRKALGRTRVKSSVCLEAYLSFCGQRGPHDP
              :    :::* : .  : *  :* *    . : :  *    *.

RGS6   LITPAEPSNPWISDDVALWDIEMS--KEPSQQRVKRWGFSFDEILKDQVG
RGS7   FLLPPDPSNPWLSDDTTFWELEAS--KEPSQQRVKRWGFGMDEALKDPVG
RGS9   IMSGCLPSNPWITDDTQFWDLNAKLVEIPTKMRVERWAFNFSELIRDPKG
RGS11 LVSGCLPSNPWISDNDAYWVMNAPTVAAPTKLRVERWGFSFRELLEDPVG
      ::    *****::*:   * ::      *:: **:**.*.: * :.*  *

RGS6  RDQFLRFLESEFSSENLRFWLAVQDLKKQPLQDVAKRVEEIWQEFLAPGA
RGS7  REQFLKFLESEFSSENLRFWLAVEDLKKRPIKEVPSRVQEIWQEFLAPGA
RGS9  RQSFQYFLKKEFSGENLGFWEACEDLKYGDQSKVKEKAEEIYKLFLAPGA
RGS11 RAHFMDFLGKEFSGENLSFWEACEELRYGAQAQVPTLVDAVYEQFLAPGA
      *  *  ** .***.*** ** * ::*:      .*   .: ::: ******

RGS6   PSAINLDSHSYEITSQNVKDGGRYTFEDAQEHIYKLMKSDSYARFLRSNA
RGS7   PSAINLDSKSYDKTTQNVKEPGRYTFEDAQEHIYKLMKSDSYPRFIRSSA
RGS9   RRWINIDGKTMDITVKGLKHPHRYVLDAAQTHIYMLMKKDSYARYLKSPI
RGS11 AHWVNIDSRTMEQTLEGLRQPHRYVLDDAQLHIYMLMKKDSYPRFLKSDM
        :*:*.:: : * :.::. **.:: ** *** ***.***.*:::*

RGS6   YQDLLLAKKK------------------GKSLAG----------------
RGS7   YQELLQAKKKSGNSMDRRTSFEKFAQNVGKSLTS----------------
RGS9   YKDMLAKAI-EPQETTKKSSTLPFMRRHLRSSPSPVILRQLEEEAKAREA
RGS11 YKALLAEAG-IPLEM--KRRVFPFTWRPRHSSPSPALLP-----------
      *: :*                             :* ..

RGS6  --------------------------------------------------
RGS7   --------------------------------------------------
RGS9   ANTVDITQPGQHMAPSPHLTVYTGTCMPPSPSSPFSSSCRSPRKPFASPS
RGS11 -----------------------------TPVEPTA-ACG----------

RGS6  --------------------------------------------------
RGS7   --------------------------------------------------
RGS9   RFIRRPSTTICPSPIRVALESSSGLEQKGECSGSMAPRGPSVTESSEASL
RGS11 --------------------------------------------------

RGS6  --------------------------------------------------
RGS7   --------------------------------------------------
RGS9   DTSWPRSRPRAPPKARMALSFSRFLRRGCLASPVFARLSPKCPAVSHGRV
RGS11 --------------------------------------------------

RGS6  ---------------KRLTGLMQS--------------------------
RGS7   ---------------KRLTSLAQS--------------------------
RGS9   QPLGDVGQQLPRLKSKRVANFFQIKMDVPTGSGTCLMDSEDAGTGESGDR
RGS11 --------------------------------------------PGGGDG

RGS6  ------------S
RGS7   ------------Y
RGS9   ATEKEVICPWESL
RGS11 VA-----------
```

Supplementary Figure 1. Sequence alignment of members of the R7-RGS protein subfamily

The amino acid sequences of human RGS6, 7, 9 and 11 were aligned using multiple sequence alignment software (Clustal Omega; European Bioinformatics Institute) to visualize the identical, similar, and dissimilar regions of the proteins. The additional 21aa region present in RGS6 and 7, but not in RGS9 and 11 proteins, are in red (LP21 region, see text).

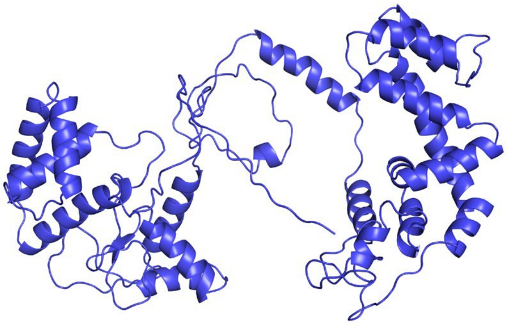

RGS6

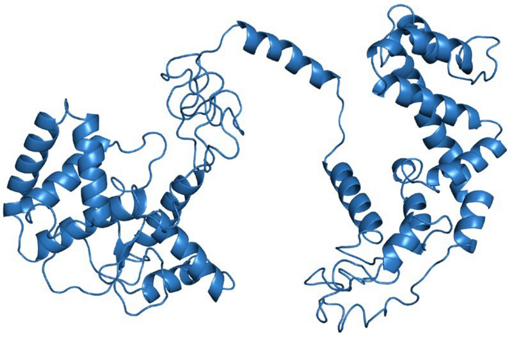

RGS7

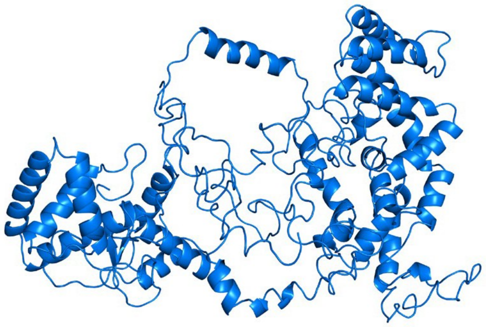

RGS9

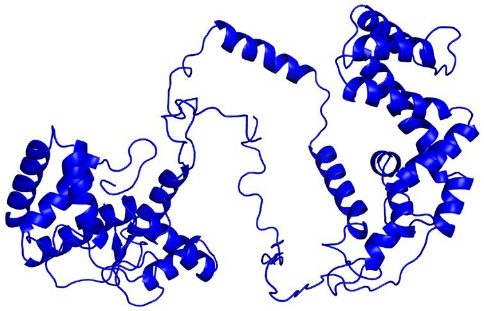

RGS11

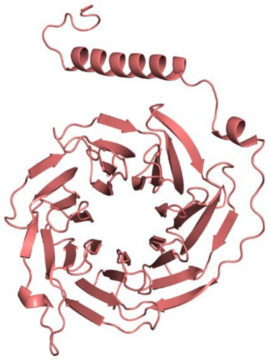

Gβ5

Supplementary Figure 2. Modeled structures of human Gβ5, RGS6, 7, 9 and 11

The predicted protein sequences of human Gβ5, RGS6, 7, 9 and 11 were analyzed by I-TASSER software to obtain the corresponding modeled structures as shown in cartoon modes (Gβ5 pink; RGS6, 7, 9 and 11, blue).

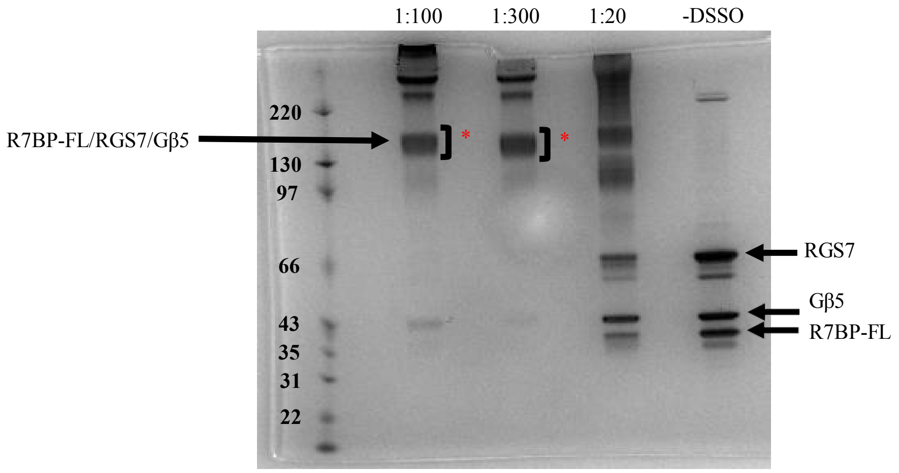

Supplementary Figure 3. SDS PAGE analysis of DSSO-cross linking results

Highly purified samples of the R7BP-FL/RGS7/Gβ5 triplex were mixed with the DSSO reagent at different molar ratios, resolved by SDS-PAGE and then stained with SimplyBlue SafeStain. The individual and fully cross-linked proteins are indicated by labeled arrows. The relative migration of protein markers (MW in kDa indicated) is shown on the left. The rightmost lane represents a sample of triplex that was not treated with DSSO.

A

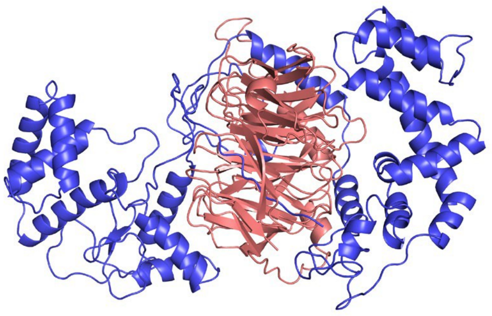

RGS6/Gβ5

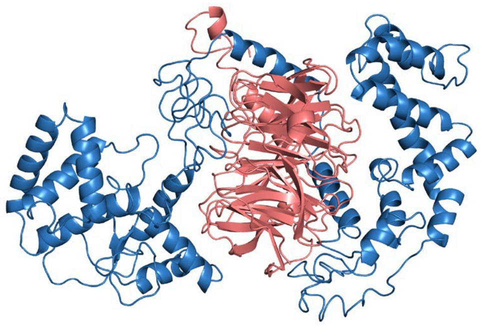

RGS7/Gβ5

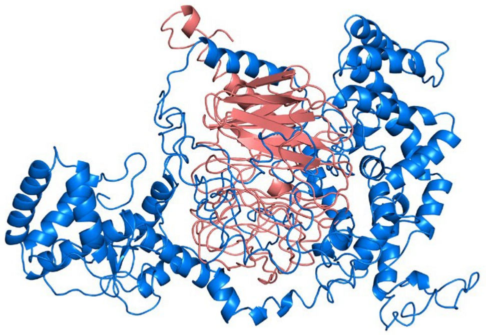

RGS9/Gβ5

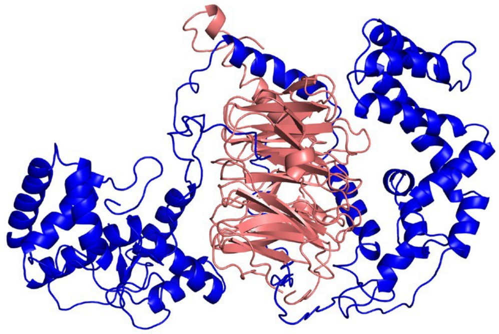

RGS11/Gβ5

B.

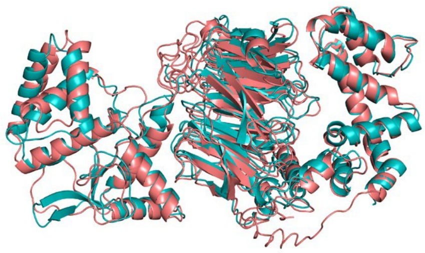

RGS7/Gβ5
RMSD = 17.1

Blue- Crystal Structure
Pink- Our Model

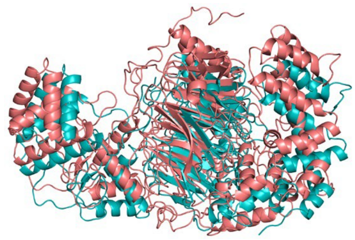

RGS9/Gβ5
RMSD = 22.3

Supplementary Figure 4. Modeled structures of human R7-RGS/Gβ5 duplexes

A. Each human R7-RGS and Gβ5 protein was compared to the mouse RGS9/Gβ5 duplex (2PBI) and RGS7/Gβ5 duplex (6N9G) structure to obtain the structures using ClusPro and CHARMM as shown (Gβ5 pink; RGS6, 7, 9 and 11, blue). **B**. The RGS7 and RGS9 models were aligned against their respective mouse crystal structures to obtain Cα carbon RMSD values as shown.

A

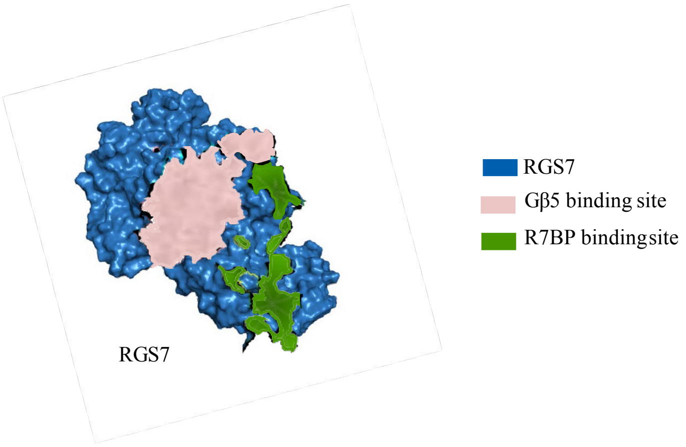

B:

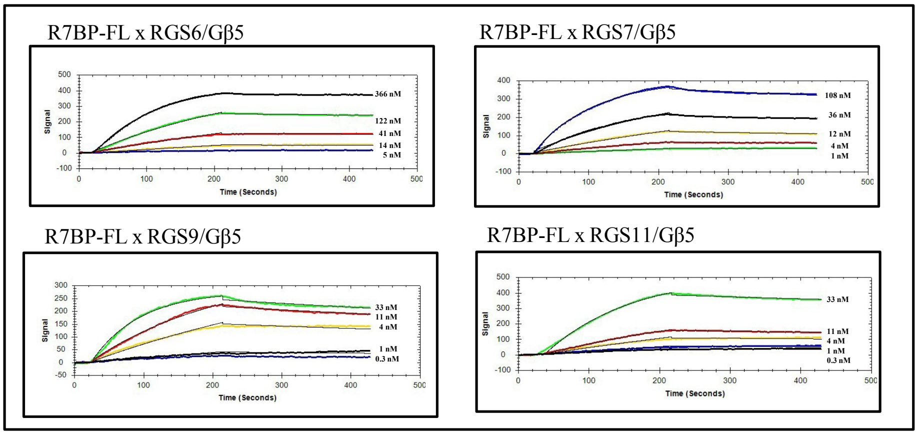

Supplementary Figure 5. R7BP/R7-RGS/Gβ5 interaction analysis

A. RGS7 interface showing the binding sites for Gβ5 (pink) and R7BP (green). **B.** Representative SPR dose-response curves at different concentrations of analyte, where R7BP-FL was used as ligand and R7-RGS/Gβ5 duplexes were used as analyte.

```
                                            34
RGS7 1      ---MAQGNNYGQTSNGVADESPNMLVYRKMEDVIARMQDEKNGIPIRTVK 47
               ..||..|          .|.|...:|:|.::..||:.:.|:.::..:
RGS9 1      MTIRHQGQQY----------RPRMAFLQKIEALVKDMQNPETGVRMQNQR 40
                                         20

RGS7 48     SFLSKIPSVFSGSDIVQWLIKNLTIEDPVEALHLGTLMAAHGYFFPISD- 96
            ..::.:|...:|||::||:::.|.|.. :||.:||..:..:||.:|:.|
RGS9 41     VLVTSVPHAMTGSDVLQWIVQRLWISS-LEAQNLGNFIVRYGYIYPLQDP 89

RGS7 97     HVLTLKDDGTFYRFQTPYFWPSNCWEPENTDYAVYLCKRTMQNKARLELA 146
            ..|.||.||:.||||||||||:..|..|:||||:||.||.::.|..||
RGS9 90     KNLILKPDGSLYRFQTPYFWPTQQWPAEDTDYAIYLAKRNIKKKGILE-- 137
                            163
RGS7 147    DYEAESLARLQRAFARKWEFIFMQAEAQAKVDKKRDKIERKILDSQERAF 196
            :||.|:...|.:....||:|:.|||:.|.:..|:|:|.:|..||.||:|:
RGS9 138    EYEKENYNFLNQKMNYKWDFVIMQAKEQYRAGKERNKADRYALDCQEKAY 187
                            155          168228       239
RGS7 197    WDVHRPVPGCVNTTEVDIKKSSRMRNPHKTRKSVYGLQNDIRSHSPTHTP 246
            |.|||..||..|..:..:   .|:.||::.:.:
RGS9 188    WLVHRCPPGMDNVLDYGL---DRVTNPNEVKVN----------------- 217
                                        216
RGS7 247    TPETKPPTEDELQQQIKYWQIQLDRHRLKMSKVADSLLSYTEQYLEYDPF 296
                :..|...::::|.|:|..|.|..:|.|.....::.|:||:...|..
RGS9 218    ----QKQTVVAVKKEIMYYQQALMRSTVKSSVSLGGIVKYSEQFSSNDAI 263
                         227         239     330
RGS7 297    LLPPDPSNPWLSDDTTFWELEAS--KEPSQQRVKRWGFGMDEALKDPVGR 344
             :....|||||::|||.||:|.|.  :.|::.||:||.|...|.::||.||
RGS9 264    MSGCLPSNPWITDDTQFWDLNAKLVEIPTKMRVERWAFNFSELIRDPKGR 313
            345                                 381
RGS7 345    EQFLKFLESEFSSENLRFWLAVEDLKKRPIKEVPSRVQEIWQEFLAPGAP 394
            :.|..||:.|||.|||.||.|.||||.....:|..:.:||::.||||||.
RGS9 314    QSFQYFLKKEFSGENLGFWEACEDLKYGDQSKVKEKAEEIYKLFLAPGAR 363
                                  417                     441
RGS7 395    SAINLDSKSYDKTTQNVKEPGRYTFEDAQEHIYKLMKSDSYPRFIRSSAY 444
            ..||:|.|:.|.|.:.:|.|.||..:.||.|||.|||.|||.|:::|..|
RGS9 364    RWINIDGKTMDITVKGLKHPHRYVLDAAQTHIYMLMKKDSYARYLKSPIY 413

RGS7 445    QELLQAKKKSGNSMDRRTSFEKFAQNVGKSLTS----KRLTSLAQSY--- 487
            :::| ||........:::|...|.:...:|..|    ::|...|::.
RGS9 414    KDML-AKAIEPQETTKKSSTLPFMRRHLRSSPSPVILRQLEEEAKAREAA 462
                   420                           451
RGS7 488    -------------------------------------------------- 487
RGS9 463    NTVDITQPGQHMAPSPHLTVYTGTCMPPSPSSPFSSSCRSPRKPFASPSR 512
                                                      505
RGS7 488    -------------------------------------------------- 487
RGS9 513    FIRRPSTTICPSPIRVALESSSGLEQKGECSGSMAPRGPSVTESSEASLD 562
                           528
RGS7 488    -------------------------------------------------- 487
RGS9 563    TSWPRSRPRAPPKARMALSFSRFLRRGCLASPVFARLSPKCPAVSHGRVQ 612

RGS7 488    -------------------------------------------------- 487
RGS9 613    PLGDVGQQLPRLKSKRVANFFQIKMDVPTGSGTCLMDSEDAGTGESGDRA 662

RGS7 488    ------------ 487
RGS9 663    TEKEVICPWESL 674
```

Supplementary Figure 6. Sequence and XL-MS analysis of RGS7 and RGS9 proteins

Alignment of human RGS7 and RGS9 amino acid sequences using Clustal Omega. The colored regions specify all cross-linked peptides that participate in inter- and/or intra- molecular interactions that were identified by XL-MS (green-RGS7, purple- RGS9, with the residue number of the initial amino acid from each cross-linked peptide indicated).

```
                                                          34
RGS7  1      MAQGNNYGQTSNGVADESP-------NMLVYRKMEDVIARMQDEKNGIPI 43
                         ..|..:|       .|...||||.|:..|||...|:.:
RGS11 1      ------------MAAGPAPPPGRPRAQMPHLRKMERVVVSMQDPDQGVKM 38

RGS7  44     RTVKSFLSKIPSVFSGSDIVQWLIKNLTIEDPVEALHLGTLMAAHGYFFP 93
             |:.:..::.||...:|||:||||.:...:.:. ||||||.::..|||.:|
RGS11 39     RSQRLLVTVIPHAVTGSDVVQWLAQKFCVSEE-EALHLGAVLVQHGYIYP 87

RGS7  94     ISD-HVLTLKDDGTFYRFQTPYFWPSNCWEPENTDYAVYLCKRTMQNKAR 142
             :.| ..|.|:.|.|.|||||||||.|........|||:||.|:.::.:.
RGS11 88     LRDPRSLMLRPDETPYRFQTPYFWTSTLRPAAELDYAIYLAKKNIRKRG- 136
                                   163
RGS7  143    LELADYEAESLARLQRAFARKWEFIFMQAEAQAKVDKKRDKIERKILDSQ 192
             .|.|||.:...||.:.....|:.:.|||..|.:..|:|.|.:|.::..|
RGS11 137    -TLVDYEKDCYDRLHKKINHAWDLVLMQAREQLRAAKQRSKGDRLVIACQ 185
                                                   228        239
RGS7  193    ERAFWDVHRPVPGCVNTTEVDIKKSSRMRNPHKTRKSVYGLQNDIRSHSP 242
             |:.:|.|:||.||..:..|....:.|...:.....||.
RGS11 186    EQTYWLVNRPPPGAPDVLEQGPGRGSCAASRVLMTKSA------------ 223

RGS7  243    THTPTPETKPPTEDELQQQIKYWQIQLDRHRLKMSKVADSLLSYTEQYLE 292
                          |..:::|:|::..|.|.|:|.|...::.||:..|...
RGS11 224    -------------DFHKREIEYFRKALGRTRVKSSVCLEAYLSFCGQRGP 260
                                                       330
RGS7  293    YDPFLLPPDPSNPWLSDDTTFWELEAS--KEPSQQRVKRWGFGMDEALKD 340
             :||.:....|||||:||:..:|.:.|.  ..|::.||:||||...|.|:|
RGS11 261    HDPLVSGCLPSNPWISDNDAYWVMNAPTVAAPTKLRVERWGFSFRELLED 310
                 345                                    381
RGS7  341    PVGREQFLKFLESEFSSENLRFWLAVEDLKKRPIKEVPSRVQEIWQEFLA 390
             ||||..|:.||..|||.|||.||.|.|:|:.....:||:.|..:::|||
RGS11 311    PVGRAHFMDFLGKEFSGENLSFWEACEELRYGAQAQVPTLVDAVYEQFLA 360
                                       417
RGS7  391    PGAPSAINLDSKSYDKTTQNVKEPGRYTFEDAQEHIYKLMKSDSYPRFIR 440
             |||...:|:||::.::|.:.:::|.||..:|||.|||.|||.||||||::
RGS11 361    PGAAHWVNIDSRTMEQTLEGLRQPHRYVLDDAQLHIYMLMKKDSYPRFLK 410
            441
RGS7  441    SSAYQELLQAKKKSGNSMDRRTSFEKFAQNVGKSLTSKRL--TSLAQSY- 487
             |..|:.|| .::|..::.:.....|......|..|..| | |.:..:.
RGS11 411    SDMYKALL---AEAGIPLEMKRRVFPFTWRPRHSSPSPALLPTPVEPTAA 457

RGS7  488    ---------- 487

RGS11 458    CGPGGGDGVA 467
```

Supplementary Figure 7. Sequence analysis of RGS7 and RGS11 proteins

Pairwise sequence alignment of human RGS7 and RGS11 using Clustal Omega. The RGS7 peptides that participate in cross-linkage are highlighted in green, with the residue number of the initial amino acid from each cross-linked peptide indicated.

A

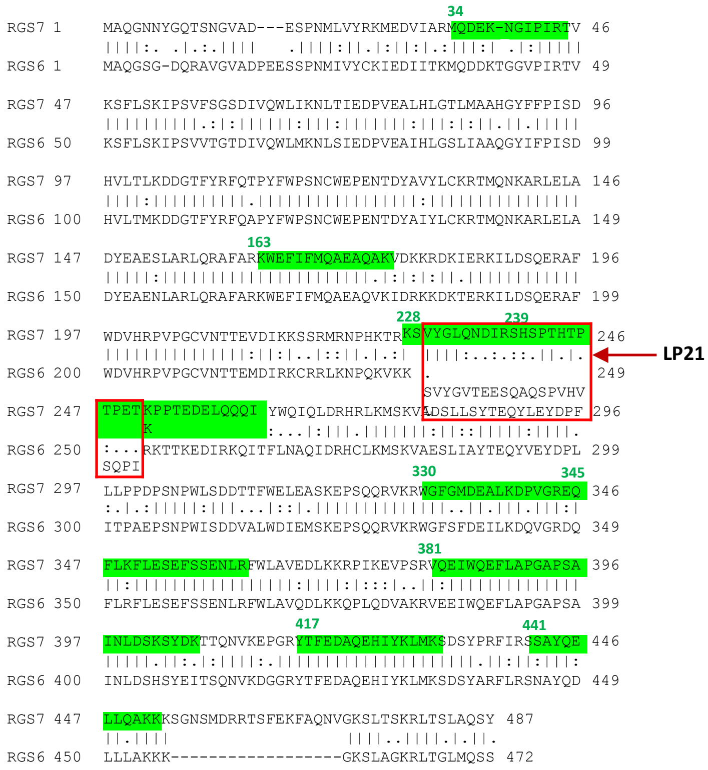

**B** LP21 peptides:	RGS6/7 full proteins:
14.3% identity	70.7% identity
57.1% similarity	83.5% similarity

Supplementary Figure 8. Sequence analysis of RGS7 and RGS6 proteins

A. Sequence alignment of human RGS6 and RGS7 proteins using Clustal Omega. All cross-linked peptides of RGS7 are highlighted in green. The 21aa region of interest is boxed in red. **B.** Analysis of this 21aa region reveals low identity and moderate similarity of the two peptide sequences, in contrast to the higher identity and similarity of the entire RGS6 and RGS7 protein sequences.

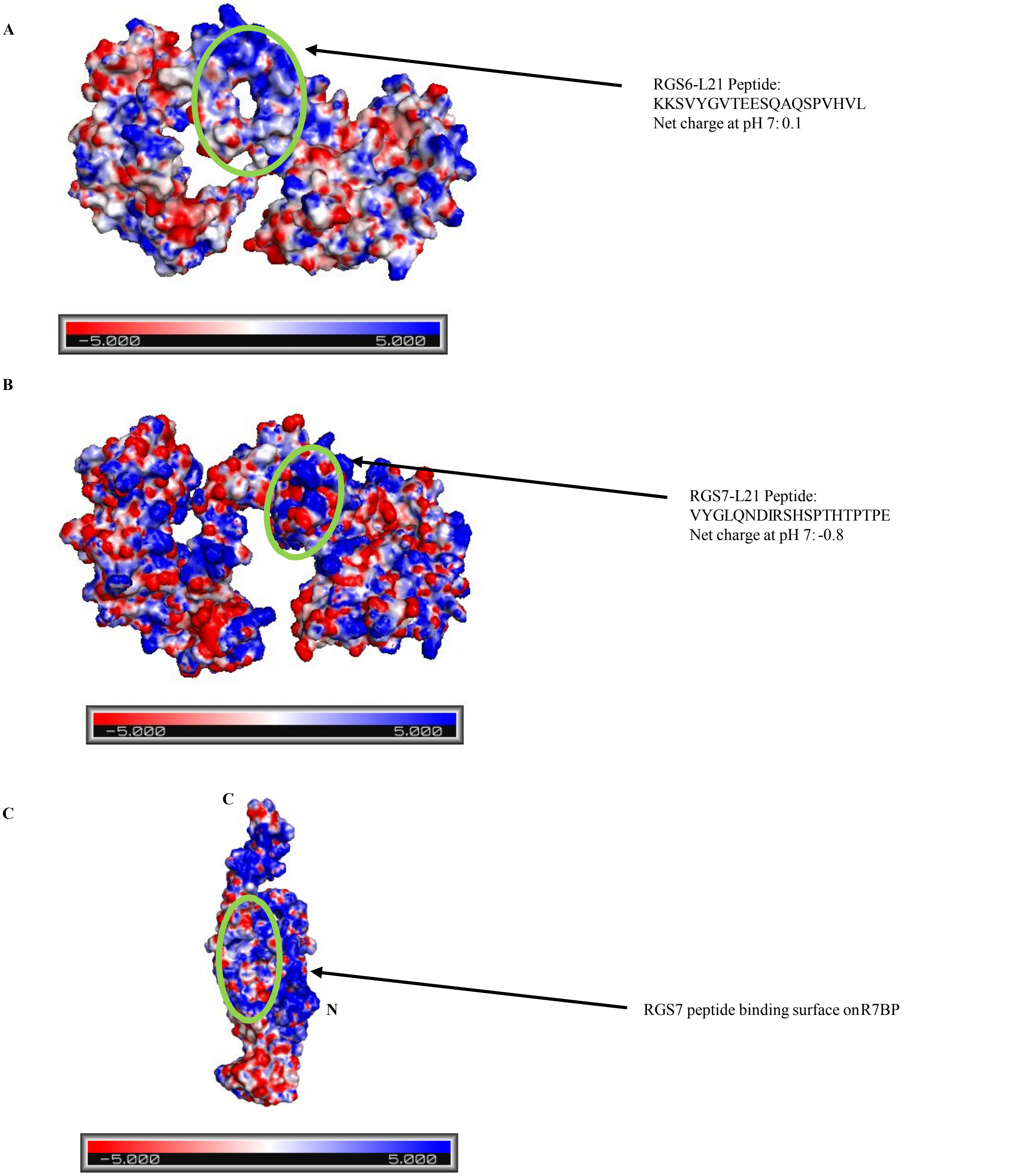

Supplementary Figure 9. Electrostatic surface potentials for RGS6, RGS7 and R7BP

The electrostatic surface potentials of RGS6 (A), RGS7 (B) and R7BP (C) generated by the PyMOL APBS plug-in are shown. The RGS6-L21 and RGS7-L21 peptide regions are highlighted by green circles. RGS6-L21 had a net charge at pH 7 of 0.1, while RGS7-L21 is -0.8 (calculated using pepcalc.com). Since R7-RGS proteins bind an R7BP interface, it is difficult to calculate the charge at the entire interaction surface.

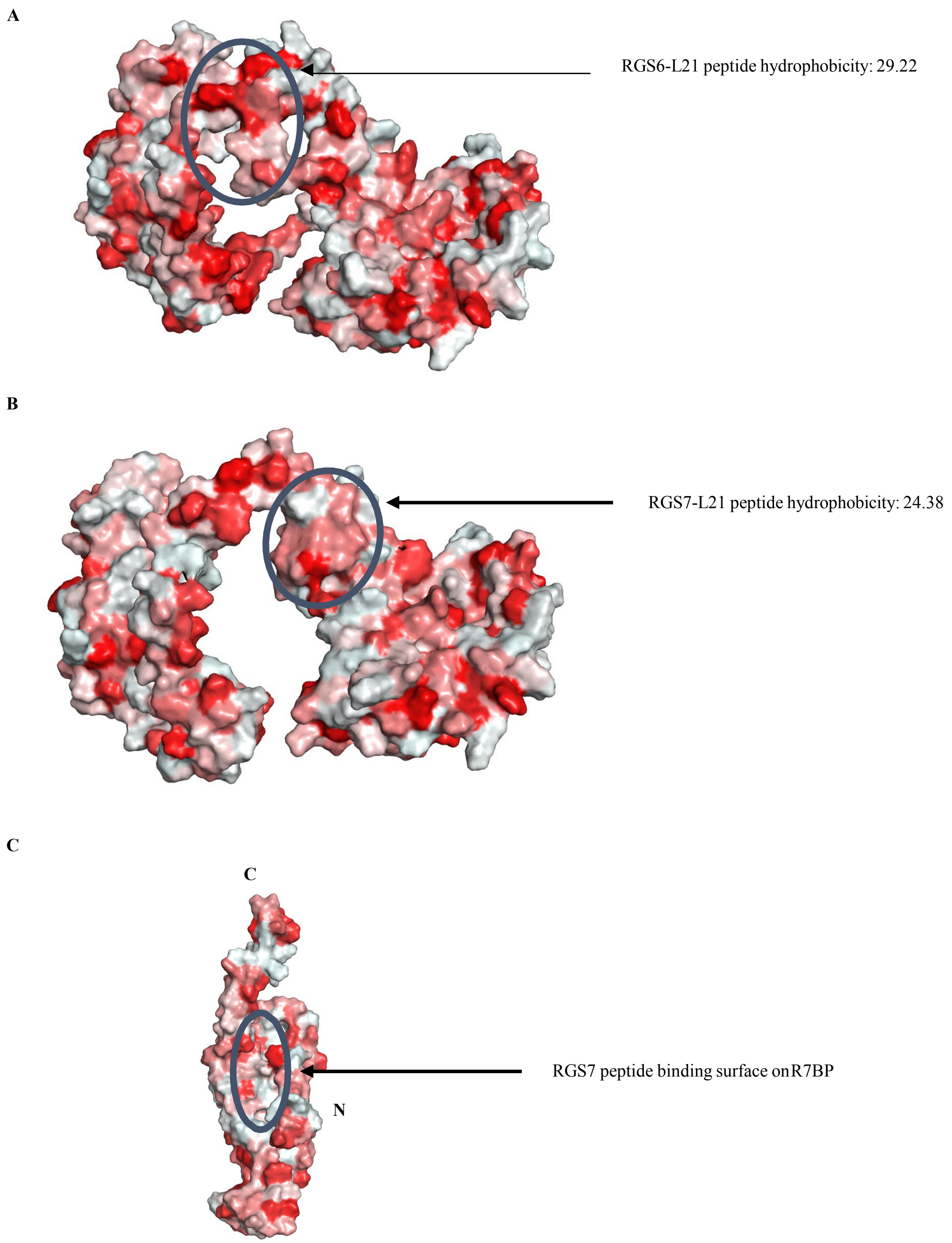

Supplementary Figure 10. Hydrophobic surfaces of RGS6, RGS7 and R7BP

The hydrophobic surfaces of RGS6 (A), RGS7 (B) and R7BP (C) were generated using PyMOL. The red color indicates hydrophobicity. The RGS6-L21 and RGS7-L21 peptide regions are shown in blue circles. RGS6-L21 had a hydrophobicity of 29.22, while RGS7-L21 was 24.38 (calculated using the Thermo Fisher Scientific peptide analyzing tool). Due to the difficulty of calculating the hydrophobicity of an interaction surface, only the binding region of R7BP is highlighted.

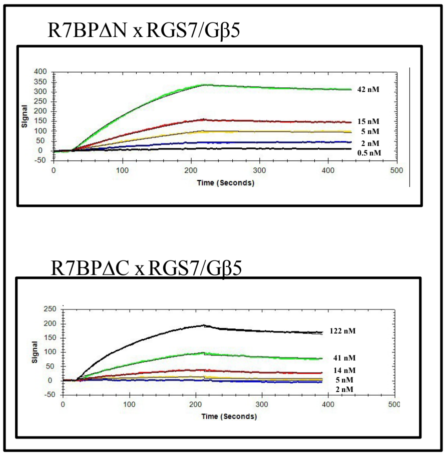

Supplementary Figure 11. SPR of R7BPΔN and R7NPΔC with RGS7/Gβ5

SPR analysis of binding affinities of the R7BP variants missing either the N- (R7BPΔN) or the C- (R7BPΔC) terminus with the RGS7/Gβ5 duplex at various concentrations

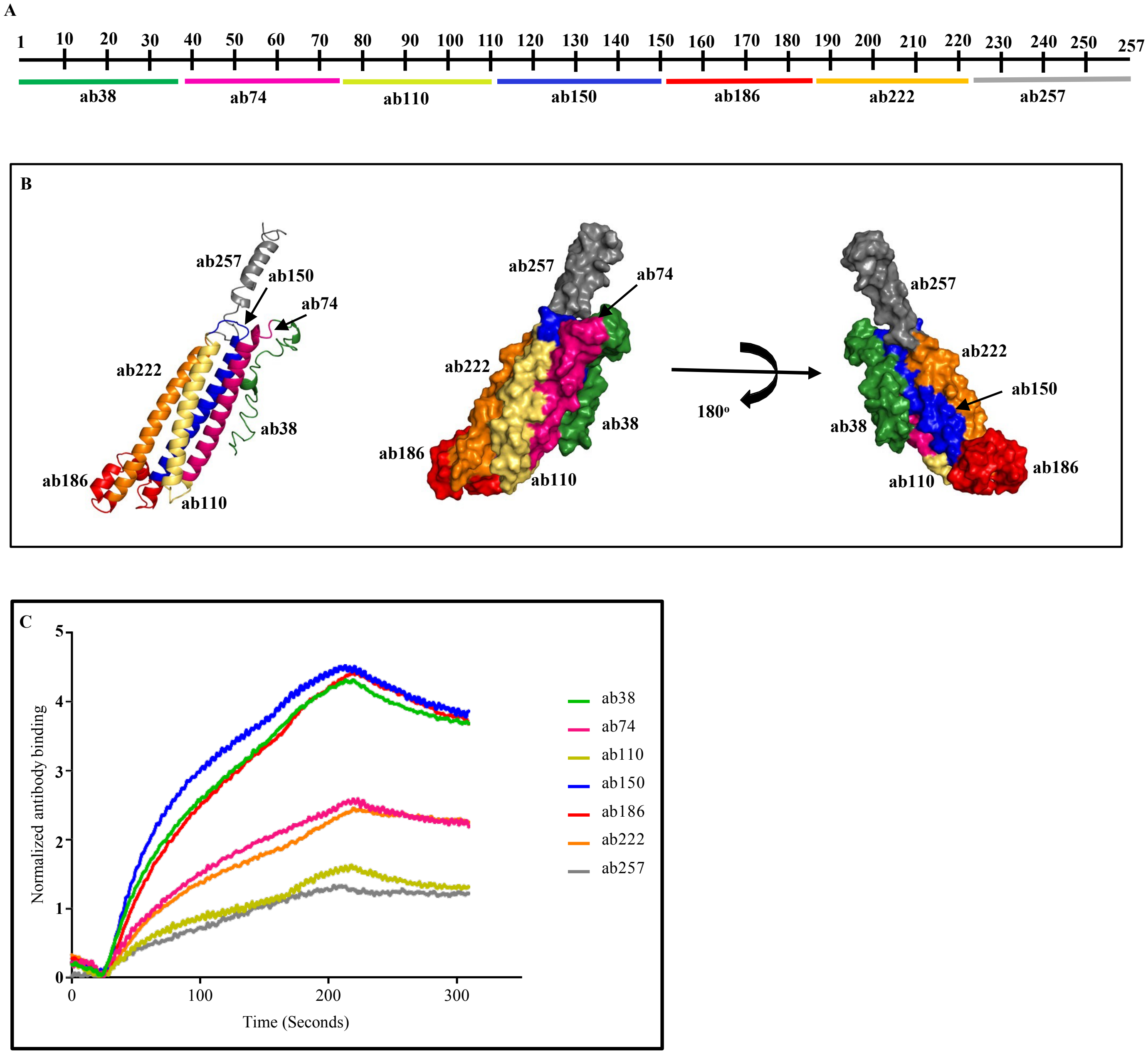

Supplementary Figure 12. Relative epitope locations for different antibodies of R7BP protein in linear and 3D models

A. The seven human R7BP peptides used for affinity purification of antibodies from the pool of total polyclonal llama antibodies are highlighted in different colors as indicated: ab38 (aa1-38, green), ab74 (aa39-74, pink), ab110 (aa75-110, yellow), ab150 (aa111-150, blue), ab186 (aa151-186, red), ab222 (aa187-222, orange) and ab257 (aa223-257, gray). **B.** The seven peptide locations were mapped onto the R7BP cartoon model and surface model (color scheme as in part A). **C.** The normalized binding affinities of each of the seven antibodies over time are shown using R7BP-FL as ligand and antibody as analyte. The binding curves were obtained by normalizing each time point to the CaSR ab negative control signal.

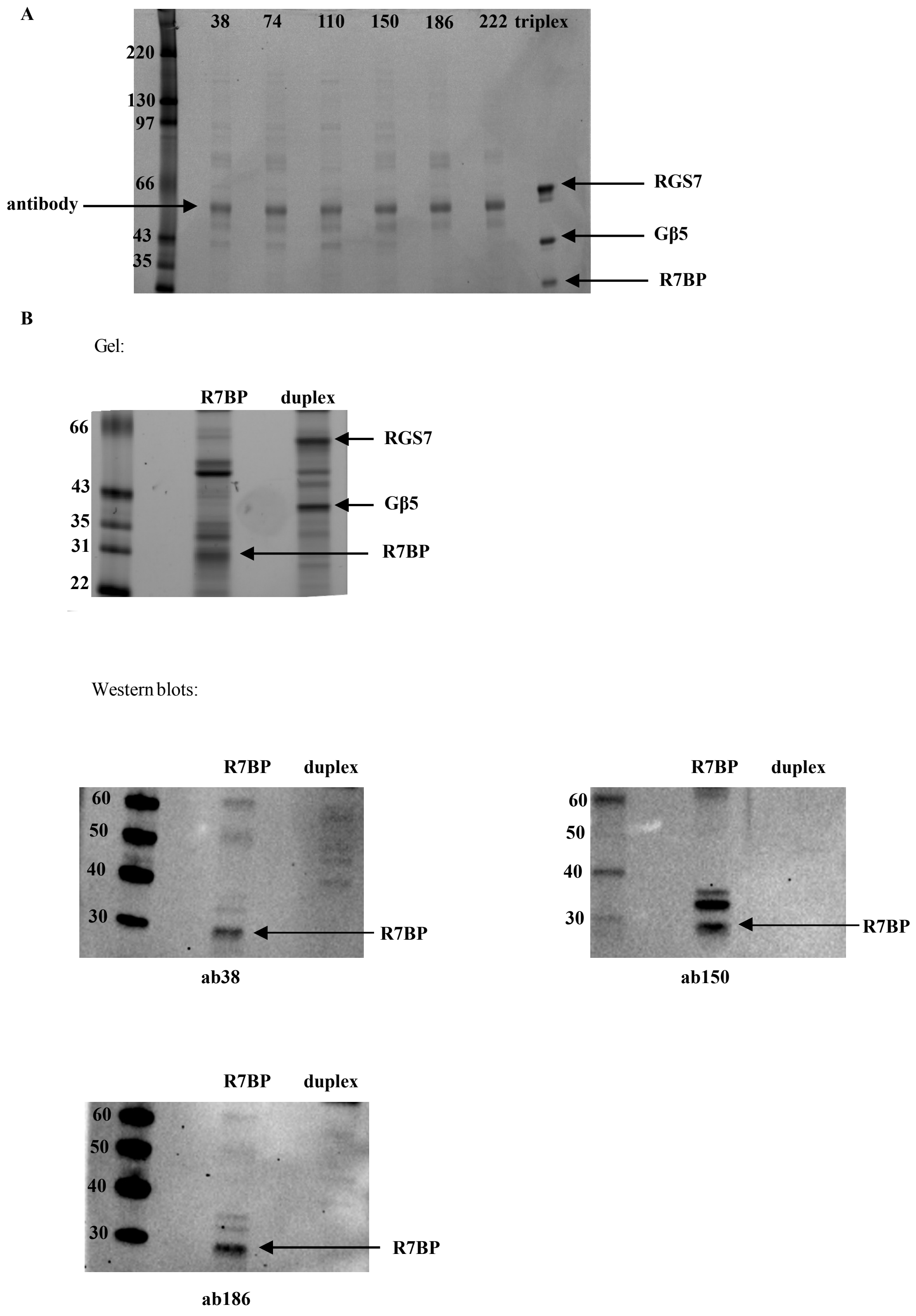

Supplementary Figure 13. Quantitation and specificity analysis of R7BP antibodies

A. Equal amounts (1 µg) of purified antibodies were run on an SDS-PAGE gel and quantified relative to a known quantity of R7BP/RGS7/Gβ5 triplex protein. **B.** R7BP (MW=29 kDa) and RGS7 (MW= 55 kDa)/HIS-Gβ5 (MW=40 kDa) were run on a gel and incubated with purified ab38, ab150 and ab186. The R7BP antibodies only show specific binding to R7BP and not the RGS7/Gβ5 duplex.

A

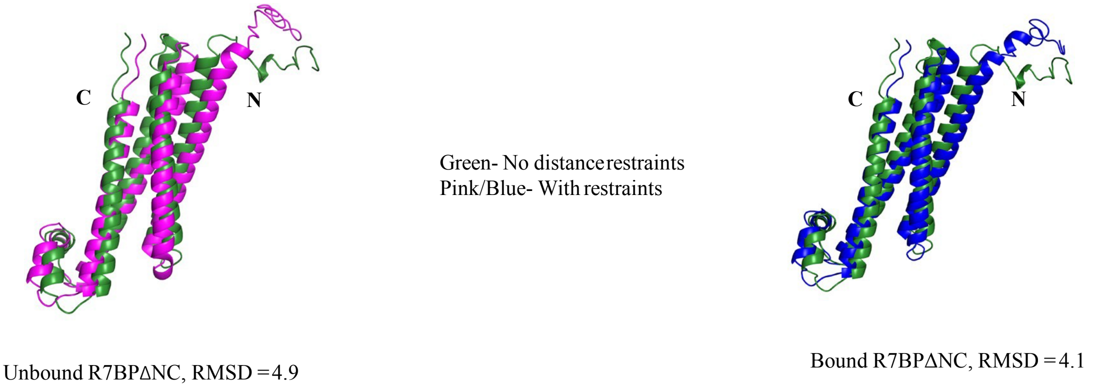

B

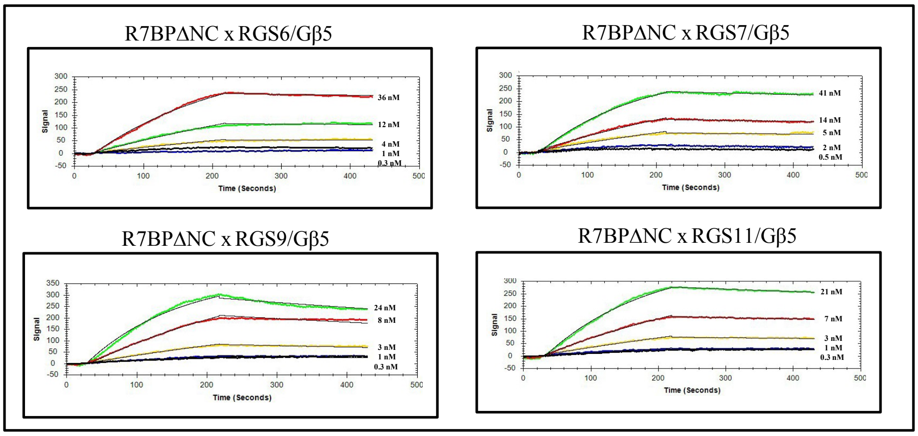

C

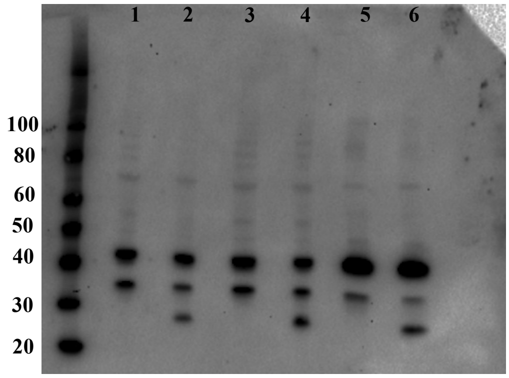

**Lane**	**Sample**
1	Lysate A
2	Lysate B
3	Supernatant A
4	Supernatant B
5	Elution A
6	Elution B

Supplementary Figure 14. R7BPΔNC structure and SPR analysis

A. R7BPΔNC models with and without distance restraints were aligned for the unbound and bound models using Cα carbon alignment. **B.** Representative SPR curves of R7BPΔNC (ligand) and R7-RGS/Gβ5 (analyte) are shown. **C.** The Western blot from Figure 6C is shown in the full uncropped form. The lysates, supernatants (after incubation with beads) and eluates from immunoprecipitation of samples A and B (see Figure 6C) are shown. Only the eluates for samples A and B are shown in Figure 6C.

Supplementary Tables

XL Proteins	1:100	1:200	1:300	Total
R7BP-FL ==X== R7BP-FL	2	1	3	6
R7BPΔNC ==X== R7BPΔNC	-	2	2	4
RGS7 ==X== RGS7	1	1	2	4
RGS9 ==X== RGS9	1	-	1	2
Gβ5 ==X== Gβ5	2	1	3	6
RGS7 ==X== R7BP-FL	1	-	1	2
RGS7 ==X== R7BPΔNC	-	1	1	2
RGS9 ==X== R7BP-FL	1	-	1	2
RGS7 ==X== Gβ5	1	1	2	4
RGS9 ==X== Gβ5	1	-	1	2

Supplementary Table 1. Summary of XL proteins and number of protein:DSSO experiments performed

The table presents all the proteins that were cross-linked, along with the number of times each experiment was performed for each protein:DSSO ratio.

Peptide	Peptide	XL Residues	NZ-NZ Distance (Å)
^{18}SSIFQIS**K**PPLQSGDWER^{35}	^{101}QAHQ**K**LAAISGPEDGEIHPEICR^{123}	K25 ==X== K105	7.3
^{18}SSIFQIS**K**PPLQSGDWER^{35}	^{218}ETMPLPL**K**NQDDSSLLNLTPYPLVR^{242}	K25 ==X== K225	21.7
^{18}SSIFQIS**K**PPLQSGDWER^{35}	^{49}ALDDC**K**MLVQEFNTQVALYR^{68}	K25 ==X== K54	13.6
^{18}SSIFQIS**K**PPLQSGDWER^{35}	^{37}GSGSESAH**K**TQR^{48}	K25 ==X== K45	19.7
^{37}GSGSESAH**K**TQR^{48}	^{49}ALDDC**K**MLVQEFNTQVALYR^{68}	K45 ==X== K54	16.3
^{37}GSGSESAH**K**TQR^{48}	^{101}QAHQ**K**LAAISGPEDGEIHPEICR^{123}	K45 ==X== K105	15.6
^{37}GSGSESAH**K**TQR^{48}	^{211}NLLS**K**LR^{217} *	K45 ==X== K215	32.4
^{37}GSGSESAH**K**TQR^{48}	^{218}ETMPLPL**K**NQDDSSLLNLTPYPLVR^{242} *	K45 ==X== K225	14.7
^{49}ALDDC**K**MLVQEFNTQVALYR^{68}	^{218}ETMPLPL**K**NQDDSSLLNLTPYPLVR^{242} *	K54 ==X== K225	18.4
^{86}AEMH**K**TR^{92}	^{93}T**K**GCEMAR^{100} *	K90 ==X== K94	8.6
^{86}AEMH**K**TR^{92}	^{18}SSIFQIS**K**PPLQSGDWER^{35} *	K90 ==X== K25	19.5
^{101}QAHQ**K**LAAISGPEDGEIHPEICR^{123}	^{86}AEMH**K**TR^{92} *	K105 ==X== K90	24.3
^{101}QAHQ**K**LAAISGPEDGEIHPEICR^{123}	^{93}T**K**GCEMAR^{100} *	K105 ==X== K94	17.8
^{101}QAHQ**K**LAAISGPEDGEIHPEICR^{123}	^{218}ETMPLPL**K**NQDDSSLLNLTPYPLVR^{242} *	K105 ==X== K225	14.7

Supplementary Table 2. XL peptides and interaction distances of unbound R7BP

Fourteen pairs of cross-linked peptides were identified in unbound R7BP. The first three colored letters of each peptide correspond to the colored peptides indicated by the arrows on each R7BP cartoon model (see Figure 1C). The linked lysine residue from each peptide is shown in bold. The asterisk represents peptide pairs that were identified in both the bound and unbound R7BP states.

Peptide	Peptide	XL Residues	NZ-NZ Distance (Å)
18SSIFQIS**K**PPLQSGDWER35	101QAHQ**K**LAAISGPEDGEIHPEICR123	K25 ==X== K105	13.9
18SSIFQIS**K**PPLQSGDWER35	218ETMPLPL**K**NQDDSSLLNLTPYPLVR242	K25 ==X== K225	23.2
18SSIFQIS**K**PPLQSGDWER35	49ALDDC**K**MLVQEFNTQVALYR68	K25 ==X== K54	5.9
18SSIFQIS**K**PPLQSGDWER35	37GSGSESAH**K**TQR48	K25 ==X== K45	14.9
37GSGSESAH**K**TQR48	49ALDDC**K**MLVQEFNTQVALYR68	K45 ==X== K54	18.4
37GSGSESAH**K**TQR48	101QAHQ**K**LAAISGPEDGEIHPEICR123	K45 ==X== K105	16.0

Supplementary Table 3. XL peptides and interaction distances of bound R7BP

Six cross-linked peptide pairs were identified in the bound R7BP state. Each pair was also identified in the bound state (see Supplementary Table 3).

Protein 1	Accessible Surface Area [ASA] (Å²)	Protein 2	ASA (Å²)	Complex	ASA (Å²)	Buried Surface Area (Å²)
RGS6/Gβ5	35073	R7BP	16222	RGS6/Gβ5/R7BP	47392	3903
RGS7/Gβ5	35652	R7BP	16222	RGS7/Gβ5/R7BP	47863	4011
RGS9/Gβ5	44805	R7BP	16222	RGS9/Gβ5/R7BP	55851	5176
RGS11/Gβ5	34434	R7BP	16222	RGS11/Gβ5/R7BP	47062	3594

Supplementary Table 4. Accessible surface area and buried surface area for modeled proteins and complexes

The accessible surface area for each R7-RGS/Gβ5 duplex, R7BP and each triplex was calculated using PyMOL. From these values, the buried surface area for each triplex was calculated.

RGS7 Peptide	R7BP Peptide	XL Residues	NZ-NZ Distance (Å)
^{34}MQDE**K**NGIPIR^{44}	^{154}**K**GKEPGGGTK^{163}	K38 ==X== K154	27.5
^{163}**K**WEFIFMQAEAQAK^{176}	^{218}ETMPLPL**K**NQDDSSLLNLTPYPLVR^{242}	K163 ==X== K225	14.0
^{228}**K**SVYGLQNDIR^{238}	^{218}ETMPLPL**K**NQDDSSLLNLTPYPLVR^{242}	K228 ==X== K225	32.8
^{228}**K**SVYGLQNDIR^{238}	^{18}SSIFQIS**K**PPLQSGDWER^{35}	K228 ==X== K25	16.1

Supplementary Table 5. RGS7-R7BP cross-linked peptides and interaction distances

Four cross-linked peptides between RGS7 and R7BP were observed. The lysine from each peptide that was cross-linked is in bold.

RGS9 Peptide	R7BP Peptide	XL Residues	NZ-NZ Distance (Å)
^{20}IEALV**K**DMQNPETGVR^{35}	^{154}**K**GKEPGGGTK^{163}	K25 ==X== K154	34.7

Supplementary Table 6. RGS9-R7BP cross-linked peptides and interaction distances

One cross-linked peptide between RGS9 and R7BP was identified.

Peptide	Peptide	XL Residues	NZ-NZ Distance (Å)
37GSGSESAH**K**TQR48	49ALDDC**K**MLVQEFNTQVALYR68	K45 ==X== K54	18.7
37GSGSESAH**K**TQR48	101QAHQ**K**LAAISGPEDGEIHPEICR123	K45 ==X== K105	17.3
49ALDDC**K**MLVQEFNTQVALYR68	93T**K**GCEMAR100	K54 ==X== K94	16.4
86AEMH**K**TR92	93T**K**GCEMAR100	K90 ==X== K94	9.7
101QAHQ**K**LAAISGPEDGEIHPEICR123	93T**K**GCEMAR100	K105 ==X== K94	12.7

Supplementary Table 7. Cross-linked peptides and interaction distances of unbound R7BPΔNC

Five cross-linked peptides were identified in unbound R7BPΔNC.

Peptide	Peptide	XL Residues	NZ-NZ Distance (Å)
37GSGSESAH**K**TQR48	49ALDDC**K**MLVQEFNTQVALYR68	K45 ==X== K54	16.4
37GSGSESAH**K**TQR48	101QAHQ**K**LAAISGPEDGEIHPEICR123	K45 ==X== K105	13.6
86AEMH**K**TR92	93T**K**GCEMAR100	K90 ==X== K94	11.7
101QAHQ**K**LAAISGPEDGEIHPEICR123	93T**K**GCEMAR100	K105 ==X== K94	11.9

Supplementary Table 8. Cross-linked peptides and interaction distances of bound R7BPΔNC

Four cross-linked intramolecular peptides in bound R7BPΔNC were observed.

RGS7 Peptide	R7BPΔNC Peptide	XL Residues	NZ-NZ Distance (Å)
228KSVYGLQNDIR238	208EMKNLLSK215	K228 ==X== K210	27.6

Supplementary Table 9. R7BPΔNC/RGS7/Gβ5 triplex crosslinked peptides between R7BPΔNC and RGS7

One cross-linked peptide between RGS7 and R7BPΔNC was identified.
