# Supplementary material for: Development of R7BP inhibitors through cross-linking coupled mass spectrometry and integrated modeling
Source: Commun Biol. 2019 Sep 13;2:338. doi: 10.1038/s42003-019-0585-1 (PMC6744478; doi:10.1038/s42003-019-0585-1)
Supplement: Supplementary file 2 — Description of Additional Supplementary Files [file 42003_2019_585_MOESM2_ESM.pdf]

Supplementary Data 1

This table presents all cross-linked peptides identified from XL-MS analysis of different proteins. The proteins analyzed, protein:DSSO ratios, individual cross-linked proteins, cross-linked peptides and cross-linked lysines are shown. The peptide spectrum matches and the number of times a XL peptide was identified from the total number of experiments are shown (see Supplementary Table 1).

Supplementary Data 2

The data in this table was collected from three different SPR experiments, normalized to the negative control antibody and compiled to yield Figure 4D.

Supplementary Data 3

The data in this table was collected from three different SPR experiments and compiled to yield Figure 4F.

Supplementary Data 4

The data in this table was compiled from three independent experiments to yield Figure 6C.
