# Supplementary material for: Development of R7BP inhibitors through cross-linking coupled mass spectrometry and integrated modeling
Source: Commun Biol. 2019 Sep 13;2:338. doi: 10.1038/s42003-019-0585-1 (PMC6744478; doi:10.1038/s42003-019-0585-1)
Supplement: Supplementary file 7 — Reporting Summary [file 42003_2019_585_MOESM7_ESM.pdf]

nature research

Corresponding author(s): William F. Simonds

Last updated by author(s): Aug 7, 2019

Reporting Summary

Nature Research wishes to improve the reproducibility of the work that we publish. This form provides structure for consistency and transparency in reporting. For further information on Nature Research policies, see Authors & Referees and the Editorial Policy Checklist .

Statistics

For all statistical analyses, confirm that the following items are present in the figure legend, table legend, main text, or Methods section.

n/a	Confirmed	
☐	☒	The exact sample size (*n*) for each experimental group/condition, given as a discrete number and unit of measurement
☐	☒	A statement on whether measurements were taken from distinct samples or whether the same sample was measured repeatedly
☐	☒	The statistical test(s) used AND whether they are one- or two-sided<br>*Only common tests should be described solely by name; describe more complex techniques in the Methods section.*
☒	☐	A description of all covariates tested
☒	☐	A description of any assumptions or corrections, such as tests of normality and adjustment for multiple comparisons
☐	☒	A full description of the statistical parameters including central tendency (e.g. means) or other basic estimates (e.g. regression coefficient) AND variation (e.g. standard deviation) or associated estimates of uncertainty (e.g. confidence intervals)
☒	☐	For null hypothesis testing, the test statistic (e.g. *F*, *t*, *r*) with confidence intervals, effect sizes, degrees of freedom and *P* value noted<br>*Give P values as exact values whenever suitable.*
☒	☐	For Bayesian analysis, information on the choice of priors and Markov chain Monte Carlo settings
☒	☐	For hierarchical and complex designs, identification of the appropriate level for tests and full reporting of outcomes
☒	☐	Estimates of effect sizes (e.g. Cohen's *d*, Pearson's *r*), indicating how they were calculated

Our web collection on statistics for biologists contains articles on many of the points above.

Software and code

Policy information about availability of computer code

Data collection	Protein modeling was performed using the online servers I-TASSER and ClusPro. Protein models were relaxed using the molecular simulation program CHARMM through NIH BioWulf. Tandem mass spectrometry (LC-MS/MS) was performed using a Dionex UltiMate 3000 rapid separation nano UHPLC system coupled online to an Orbitrap Fusion Lumos tribrid mass spectrometer for XL-MS experiments. SPR data were collected using OpenSPR software available with the Nicoya OpenSPR instrument.
Data analysis	Protein models were visualized using PyMOL (version 1.8.2.3). Raw data files from LC-MS were analyzed using a Proteome Discoverer software package from Thermo. Cross-linked peptides were generated using a custom script. SPR data was analyzed using TraceDrawer software available from Nicoya LifeSciences. Western blots were analyzed using ImageJ (1.51n).

For manuscripts utilizing custom algorithms or software that are central to the research but not yet described in published literature, software must be made available to editors/reviewers. We strongly encourage code deposition in a community repository (e.g. GitHub). See the Nature Research guidelines for submitting code & software for further information.

Data

Policy information about availability of data

All manuscripts must include a data availability statement. This statement should provide the following information, where applicable:

- Accession codes, unique identifiers, or web links for publicly available datasets
- A list of figures that have associated raw data
- A description of any restrictions on data availability

All cross-linking mass spectrometry data were uploaded to PeptideAtlas.org and available for public viewing on 12/01/2019 or upon publication of paper. Data is available at: www.peptideatlas.org/PASS/PASS01391

October 2018

Field-specific reporting

Please select the one below that is the best fit for your research. If you are not sure, read the appropriate sections before making your selection.

- [x] Life sciences
- [ ] Behavioural & social sciences
- [ ] Ecological, evolutionary & environmental sciences

For a reference copy of the document with all sections, see nature.com/documents/nr-reporting-summary-flat.pdf

Life sciences study design

All studies must disclose on these points even when the disclosure is negative.

Sample size	Cross-linking was performed at two protein:DSSO ratios, which is sufficient to cross-link all peptides. SPR experiments to measure binding constants were conducted twice using 3-5 analyte concentrations, as recommended by the Nicoya team. SPR experiments to assess binding and blocking of antibodies was conducted in triplicate to obtain statistically relevant sampling size. The in vivo competition assay was performed in triplicate for this reason also.
Data exclusions	No data was excluded from the analysis.
Replication	All experiments were replicated and successful.
Randomization	Randomization was not relevant to our study because we experimented on specific proteins.
Blinding	Blinding was not relevant to our study because we worked with specific proteins.

Reporting for specific materials, systems and methods

We require information from authors about some types of materials, experimental systems and methods used in many studies. Here, indicate whether each material, system or method listed is relevant to your study. If you are not sure if a list item applies to your research, read the appropriate section before selecting a response.

Materials & experimental systems

n/a	Involved in the study
[ ]	[x] Antibodies
[ ]	[x] Eukaryotic cell lines
[x]	[ ] Palaeontology
[x]	[ ] Animals and other organisms
[x]	[ ] Human research participants
[x]	[ ] Clinical data

Methods

n/a	Involved in the study
[x]	[ ] ChIP-seq
[x]	[ ] Flow cytometry
[x]	[ ] MRI-based neuroimaging

Antibodies

Antibodies used	All antibodies were made in-house. Two were purified from rabbit serum and the other eight were purified from llama serum. Details of purification and quantification are provided in the text.
Validation	Antibodies were validated against purified protein and referenced against known molecular weight.

Eukaryotic cell lines

Policy information about cell lines

Cell line source(s)	Expi293 cells were purchased from Thermo Fisher.
Authentication	Cell line was not authenticated after purchase from Thermo Fisher.
Mycoplasma contamination	Cell line was not tested for mycoplasma contamination.
Commonly misidentified lines (See ICLAC register)	*Name any commonly misidentified cell lines used in the study and provide a rationale for their use.*
